# Arteriovenous Malformations in Proximal Part of Ileum: A Case Report

**DOI:** 10.31729/jnma.6929

**Published:** 2021-07-31

**Authors:** Saad Saeed, Sidra Naz, Abbas Iqbal, Maryam Irfan, Shahab Khan, Vikash Jaiswal, Asmita Neupane

**Affiliations:** 1 Hayatabad Medical Complex, Pakistan; 2 University of Health Science, Lahore, Pakistan; 3 Ayub Teaching Hospital, Pakistan; 4 Hayatabad Medical Complex, Pakistan; 5 Khyber Teaching Hospital, Peshawar, Pakistan; 6 AMA School of Medicine, Makati, Philippines; 7 Kathamndu Medical College Teaching Hospital, Sinamangal, Kathmandu, Nepal

**Keywords:** *arteriovenous malformation*, *ileum*, *enteroscopy*

## Abstract

Arteriovenous malformations in the ileum are a rare cause of gastrointestinal bleeding in young adults with few reported cases and pose difficulty in diagnosing. They usually present with chronic gastrointestinal bleed. A 30-year-old woman presented with an acute episode of hematochezia with a history of intermittent melena for 1.5 years. Complete blood count revealed a low hemoglobin level of 3.5g/dl and hypochromic microcytic anemia. Oesophago-gastro-duodenoscopy was normal; however, a colonoscopy revealed the terminal ileum and colon filled with blood. Computed tomography-Angiogram showed local intraluminal contrast extravasation in the ileum. Explorative laparotomy and on-table enteroscopy were performed identifying a small elevated, pigmented, and eroded mucosa (5 to 6mm) in proximal ileum; resection and primary anastomosis were performed. The patient was followed after surgical resection and her symptoms improved dramatically with no additional episodes of melena and with the normalization of hemoglobin.

## INTRODUCTION

Small intestine bleeding is a rare cause of gastrointestinal (GI) bleed; however, it is common cause of obscure gastrointestinal bleed (OGIB).^1^ Small intestinal GI bleeds are difficult to locate making their diagnosis often exceedingly difficult. Arteriovenous malformations are an important subtype of these vascular malformations that rarely occur in the ileum with difficulty to diagnose.^2^ Endoscopy, colonoscopy, and computed tomography (CT)-Angiogram can be inconclusive, and patient often needs enteroscopy or capsule endoscopy to localize the site of the lesion.^3^ Treatment options depend on size, site, and status of the lesion (actively bleeding or covered by a clot). Embolization, sclerotherapy, and surgical resection of the segment were among the treatment modalities of choice.^1^

## CASE REPORT

A 30-year-old woman presented to Accident and Emergency department with an acute episode of fresh bleed per rectum for three days. She also complains of black colored stool, palpitations, fatigue, and shortness of breath for one and a half year. She had no significant comorbidities, no history of acid peptic ulcer disease, NSAIDS use, chronic liver disease, anticoagulant, or anti-platelet drug usage. Blood investigations on admission showed hemoglobin (Hb) of 3.5g/dl, HCT 11.5% with a microcytic hypochromic anemia picture on peripheral smear. Other hematological and biochemical investigations were within the normal range.

The patient was resuscitated in emergency department. During her hospital stay, she received 9 pints of packed red blood cell (PRBCs) and 3 pints of fresh frozen plasma (FFP) which increased her Hb to 10.2g/dl. OGD was unremarkable, however colonoscopy showed that the terminal ileum and colon contained fresh and altered blood with normal visible mucosa. Source of bleed could not be identified. The patient was then referred for CT mesenteric angiogram which revealed focal intraluminal contrast extravasation in ileal loop.

**Figure 1 A, B.**
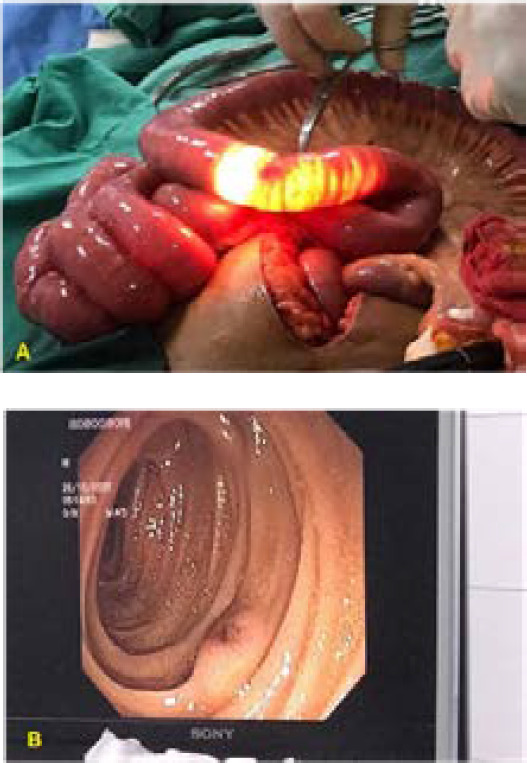
On-table laparotomy showing hyperemic area in the wall of bowel and enteroscopy (showing a bright red elevated area in the proximal ileum).

Despite all these investigations, the source of bleed could not be pin-pointed, and the patient's condition was not improving with constant melena (6 episodes per day) and decrease in Hb level. An explorative laparotomy and an on-table enteroscopy were then performed in the best interest of the patient. Preoperatively, no gross pathology (ulcers, mass, diverticula) of the small or large intestine were noticed. Enteroscopy was done in search for any intraluminal foci of bleed. Enteroscopy showed fresh and altered blood starting from ileum up to ileocecal junction (Figure 1). A small elevated, pigmented, and eroded mucosa (5-6mm) was also identified in the proximal ileum.

That part of ileum was resected, and primary anastomosis was done. A sample was sent for biopsy which revealed submucosal based vessels (arterioles and venules) with a tortuous course. The patient was followed for a few months after the surgery and her condition was improved significantly with no episode of melena.

## DISCUSSION

AVM cases in the ileum are a rare cause of small bowel GI bleeding and occur in approximately only 5.5% of cases.^4^ The pathogenesis of AVMs is not clearly understood, however, acquired AVMs are thought to arise through a “mechanical theory” which is due to increase bowel wall pressure and chronic hypoxia leading to submucosal vein obstruction.^5^ Moreover, the occurrence of AVMs in men and women is equal with no predilection for race.^6^

There exists a classification system developed by Moore, et al. that divides intestinal AVMs into 3 types based on their location, age of patient, and family history.^7^ This classification includes Type 1 (acquired AVMs in the elderly, generally small in size), Type 2 (congenital AVMs in young adults, usually large in size) and Type 3 (AVMs in patients with hereditary hemorrhagic telangiectasia.^7^ According to this classification, the patient is thought to have presented with a type 2 AVM.

The most common presentation of patients with a small bowel AVM is hematemesis, melena, or anemia^2^ as seen in this patient. They are rarely fatal, however, massive upper GI bleeding with hemodynamic instability might occur.^6^ They also can present as obscure chronic GI bleeding. Obscure chronic GI bleed requires a multidisciplinary approach for diagnosis. In cases of chronic GI blood loss and anemia, different imaging modalities such as include OGD, small bowel radiography, radionuclide imaging, colonoscopy, and capsule endoscopy should be utilized prior to exploratory laparotomy as initial modes of investigation.^3^ Intraoperative enteroscopy (IOE) is the last resort and gold standard for evaluation of OGIB. IOE is indicated when small bowel vascular lesions are difficult to localize with other modalities, cannot be treated by endoscopic or angiographic embolization and require surgical resection or when the patient condition does not allow for non-invasive diagnostic evaluations.^8^ In this case, the patient's lesion could not be identified with any imaging modality and as such required IOE.

The treatment options of symptomatic intestinal vascular malformations include intravascular embolization and surgical resection.^1^ However, in cases of intravascular embolization, the recurrence rate is more common.^9^ Since our patient was young, surgical resection was the preferred treatment due to the lower recurrence rate.
